# Epidemiology, prognostic factors, and treatment of head and neck mucoepidermoid carcinoma: Analysis of the surveillance, epidemiology, and end results database^☆^

**DOI:** 10.1016/j.bjorl.2024.101450

**Published:** 2024-06-08

**Authors:** Lin Gui, Yiming Zhu, Ye Zhang, Le Tang, Jiarui Yao

**Affiliations:** a National Cancer Center/National Clinical Research Center for Cancer/Cancer Hospital, Chinese Academy of Medical Sciences & Peking Union Medical College, Department of Medical Oncology, Beijing, China; b National Cancer Center/National Clinical Research Center for Cancer/Cancer Hospital, Chinese Academy of Medical Sciences & Peking Union Medical College, Department of Head and Neck Surgical Oncology, Beijing, China; c National Cancer Center/National Clinical Research Center for Cancer/Cancer Hospital, Chinese Academy of Medical Sciences & Peking Union Medical College, Department of Radiation Oncology, Beijing, China

**Keywords:** Mucoepidermoid carcinoma, Head and neck, Overall survival, Cancer-specific survival, Treatment modality

## Abstract

**Objectives:**

To investigate prognostic factors in patients with head and neck Mucoepidermoid Carcinoma (MEC), especially the impact of treatment modalities on survival.

**Methods:**

Patients with primary head and neck MEC between 2000 and 2015 were obtained from the Surveillance, Epidemiology, and End Results (SEER) database. Prognostic factors related to Overall Survival (OS) and Cancer-Specific Survival (CSS), as well as the impact of treatments, were evaluated by multivariable Cox regression analysis.

**Results:**

We identified 2692 patients diagnosed with head and neck MEC, of whom 1397 (51.89%) had a parotid gland primary, 569 (22.14%) died, and 341 (12.67%) died of MEC. Older age (≥53 years), males, unmarried, lower income, tumor site in other head and neck areas, higher tumor grade, larger tumor size, and higher stage were related to poorer OS and CSS. Patients who did not undergo surgery (HR = 3.20, 95% CI 2.45‒4.18) had worse OS, while no significant difference was detected between partial and total organ excision on patients’ OS (*p* = 0.729). For combination therapy, patients who received radiotherapy only (HR = 3.21, 95% CI 2.27–4.53) or no surgery and no radiotherapy (HR = 2.59, 95% CI 1.83–3.67) were correlated with worse OS (vs. surgery only), but no significant difference was detected between surgery only and surgery combined with radiotherapy on patients’ OS (*p* = 0.218). For CSS, the corresponding results were consistent with OS.

**Conclusion:**

Surgical resection only may be a better survival option for head and neck MEC.

## Highlights

• Older age and males were correlated with poorer OS and CSS in patients with head and neck mucoepidermoid carcinoma (MEC).
• No surgery was associated with poor OS.
• No significant difference was detected between partial and total organ excision on patients’ OS.
• There was no significant difference between surgery only and surgery combined with radiotherapy on patients’ OS.
• Surgical resection only may be a better survival option for head and neck MEC.

## Introduction

Mucoepidermoid Carcinoma (MEC) is a malignant tumor of glandular epithelium. MEC is histologically composed of three different types of cells: epidermoid squamous cells, mucinous cells, and intermediate cells.^1^ Histologic grading of MEC is usually divided into low-, intermediate-, and high-grade.^2^ As histologic grading increased, MEC became more infiltrative and less well demarcated.^2^ The head and neck are the main primary sites of MEC, with parotid MEC being the most common.^3^ Furthermore, the prevalence of MEC is higher in females than in males.^4^, ^5^ Histologic grading and staging are major factors influencing the treatment and prognosis of MEC.^6^, ^7^

The primary treatment for MEC is surgical resection.^2^ Surgical resection of disease-free margins can achieve good therapeutic results for low or intermediate grade MEC, whereas high grade tumors are usually aggressive with poor regional control and surgical resection alone may not be sufficient.^8^, ^9^, ^10^ Adjuvant radiotherapy is recommended for MEC patients after surgery.^11^, ^12^ However, the impact of adjuvant radiotherapy on survival in head and neck MEC is inconsistent.^13^, ^14^ In addition, although the benefit of surgical resection on survival in head and neck MEC is well established, the impact of the scope of surgical resection (partial/total excision) on survival remains unclear. Previous studies have shown that the impact of primary tumor site on head and neck MEC survival is also controversial.^15^, ^16^ Patel et al. found that salivary gland MEC had significantly better 5-year cancer-specific survival than sinonasal MEC.^15^ A small sample cohort study found no impact of different primary sites on survival in head and neck MEC.^16^ The epidemiologic characteristics of head and neck MEC, especially the factors influencing prognosis, are not sufficiently understood.

Thus, this study was to explore the factors correlated with the prognosis of head and neck MEC and to analyze the impact of treatment modalities on patient prognosis based on the nationally representative Surveillance, Epidemiology, and End Results (SEER) database.

## Methods

### Study design and data source

Data were extracted from the SEER database (17 registries, Nov 2021 Sub [2000–2019]) between 2004 and 2015. The SEER database is a national program that publishes cancer incidence and survival data and covers approximately 48% of the U.S. population (https://seer.cancer.gov/). Data collected in the SEER database include demographics, tumor morphology, tumor site, stage, treatment, and vital status. Patients diagnosed with primary head and neck MEC were included. The excluded criteria were as follows: 1) <18 years of age at diagnosis; 2) Diagnosis of MEC from autopsy or death certificate; 3) Survival time < 1 month; 4) Patients with unclear surgical information; 5) Patients with a T-stage of T0; and 6) Patients with missing follow-up information. MEC was identified using the histological classification code 8430/3 of the International Classification of Disease for Oncology, Third Edition (ICD-O-3). Since the data in this study are de-identified publicly available data, this retrospective cohort study was exempted from the institutional ethical committee by our hospital.

### Outcomes

Overall Survival (OS) and Cancer-Specific Survival (CSS) were used as outcomes. Death from any cause occurring between diagnosis and the end of follow-up was OS. Death from MEC between diagnosis and the end of follow-up was CSS. Survival time was the time from diagnosis to death, or to loss to follow-up, or to the end of administrative follow-up (December 31, 2019).

### Data collection

Data were extracted from the SEER database using SEER*STAT version 8.4.0. Data on patients were collected including age, sex, income, race (White, Black, others, unknown), marital status, primary site (parotid gland, other major salivary glands, palate, other oropharyngeal areas, and other head and neck areas), tumor size, TNM stage, tumor grade (I [well-differentiated], II [moderately differentiated], III [poorly differentiated], IV [undifferentiated], unknown), American Joint Committee on Cancer (AJCC) stage, chemotherapy (no/unknown, yes), surgery (no, partial organ excision, total organ excision, unknown type of surgery), radiation (no, yes), combination therapy (no surgery and no radiotherapy, surgery only, radiotherapy only, surgery combined with radiotherapy), and survival time. For combination therapy, only surgery and radiotherapy were considered in this study due to few patients received chemotherapy.

### Statistical analysis

Survivors and non-survivors in head and neck MEC patients were characterized. Continuous data were presented as mean ± Standard Deviation (SD) or median with quartiles (Q1, Q3) and categorical data were presented as numbers with percentages. Differences in continuous data were compared using the *t*-test or Wilcoxon rank-sum test, and differences in categorical data were compared using the χ^2^ test or Fisher’s exact test.

Kaplan-Meier (KM) curves were utilized to explore OS and CSS in patients with different age (<53 years, ≥53 years), sex (female, male), tumor primary site (parotid gland, other major salivary glands, palate, other oropharyngeal areas, and other head and neck areas), tumor grade (I, II, III, IV, unknown), and AJCC stage (I, II, III, IV, unknown), and the log-rank test was applied. The covariates of OS and CSS were screened using a univariable Cox proportional hazard model. Factors affecting OS and CSS were analyzed using a multivariable Cox proportional hazard model. Moreover, the effects of treatments on survival were analyzed. Subgroup analyses were further used to evaluate the impact of treatments on OS and CSS in different subgroup populations. Due to the heterogeneity of the MEC, patients were divided into two groups based on tumor grade (tumor grade I/II and tumor grade III/IV) to analyze the effect of treatment on survival in different tumor primary sites. Hazard Ratio (HR) with 95% Confidence Interval (95% CI) were estimated. Statistical analyses were completed using SAS 9.4 (SAS Institute Inc., Cary, NC, USA) and R version 4.2.2 was used for forest plotting. Statistical significance was set as a *p*-value < 0.05.

## Results

### Patient characteristics

**Figure 1:**
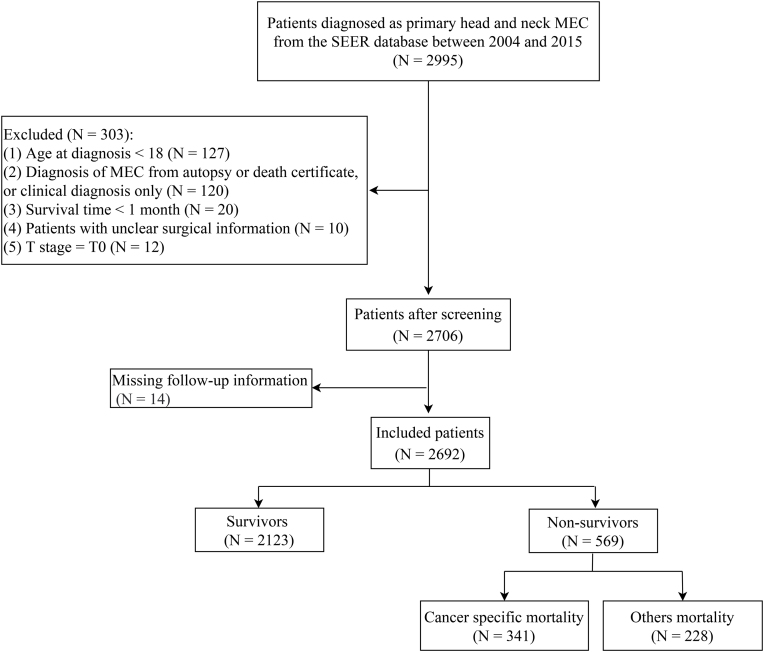
Flow chart for patients. MEC, Mucoepidermoid Carcinoma; SEER, Surveillance, Epidemiology, and End Results database.

We identified 2995 patients with primary head and neck MEC from the SEER database 2004‒2015. Finally, 2692 eligible patients were enrolled in the study (Fig. 1). Table 1 presents the characteristics of these 2692 patients. The median age was 53.00 (39.00, 65.00) years and 1444 (53.64%) patients were female. For the primary tumor site, 1397 (51.89%) patients were in the parotid gland, 260 (9.66%) patients in other major salivary glands, 396 (14.71%) patients in the palate, 556 (20.65%) patients in other oropharyngeal areas, and 83 (3.08%) patients in other head and neck areas. Only 162 (6.02%) patients received chemotherapy. For surgery, 1615 (59.99%) patients received partial organ excision, 803 (29.83%) patients received total organ excision or radical excision, 60 (2.23%) patients received an unknown type of surgery, and 214 (7.95%) patients did not receive surgery. For combination therapy, 1609 (59.77%) patients received surgery only, 79 (2.93%) patients received radiotherapy only, 869 (32.28%) patients received surgery combined with radiotherapy, and 135 (5.01%) patients received neither surgery nor radiotherapy. At the end of the current follow-up, 2123 (78.86%) patients were survivors, 569 (22.14%) patients died, and 341 (12.67%) patients died of MEC. The median follow-up time was 93.00 (57.00, 136.00) months.

**Table 1:** Characteristics of patients with head and neck Mucoepidermoid Carcinoma (MEC).

Variables	Total (n = 2692)	Survivors (n = 2123)	Non-survivors (n = 569)	*p*
Age, years, M (Q_1_, Q_3_)	53.00 (39.00, 65.00)	49.00 (36.00, 60.00)	70.00 (58.00, 81.00)	<0.001
Age, n (%)				<0.001
<53 years	1323 (49.15)	1228 (57.84)	95 (16.70)	
≥53 years	1369 (50.85)	895 (42.16)	474 (83.30)	
Sex, n (%)				<0.001
Female	1444 (53.64)	1220 (57.47)	224 (39.37)	
Male	1248 (46.36)	903 (42.53)	345 (60.63)	
Race, n (%)				<0.001
White	2002 (74.37)	1534 (72.26)	468 (82.25)	
Black	310 (11.52)	243 (11.45)	67 (11.78)	
Others	314 (11.66)	281 (13.24)	33 (5.80)	
Unknown	66 (2.45)	65 (3.06)	1 (0.18)	
Marital status, n (%)				<0.001
Married	1485 (55.16)	1204 (56.71)	281 (49.38)	
Unmarried	974 (36.18)	718 (33.82)	256 (44.99)	
Unknown	233 (8.66)	201 (9.47)	32 (5.62)	
Income, n (%)				0.008
≥75,000$	876 (32.54)	717 (33.77)	159 (27.94)	
<75,000$	1816 (67.46)	1406 (66.23)	410 (72.06)	
Primary site, n (%)				<0.001
Parotid gland	1397 (51.89)	1060 (49.93)	337 (59.23)	
Other major salivary glands	260 (9.66)	191 (9.00)	69 (12.13)	
Palate	396 (14.71)	368 (17.33)	28 (4.92)	
Other oropharyngeal areas	556 (20.65)	455 (21.43)	101 (17.75)	
Other head and neck areas	83 (3.08)	49 (2.31)	34 (5.98)	
Tumor grade, n (%)				<0.001
I	680 (25.26)	631 (29.72)	49 (8.61)	
II	1217 (45.21)	1075 (50.64)	142 (24.96)	
III	278 (10.33)	130 (6.12)	148 (26.01)	
IV	260 (9.66)	107 (5.04)	153 (26.89)	
Unknown	257 (9.55)	180 (8.48)	77 (13.53)	
Tumor size, mm, M (Q_1_, Q_3_)	20.00 (13.00, 40.00)	20.00 (12.00, 32.00)	34.00 (21.00, 55.00)	<0.001
Tumor size, n (%)				<0.001
<1 cm	338 (12.56)	322 (15.17)	16 (2.81)	
1−2 cm	852 (31.65)	764 (35.99)	88 (15.47)	
2−4 cm	858 (31.87)	626 (29.49)	232 (40.77)	
≥4 cm	284 (10.55)	132 (6.22)	152 (26.71)	
Unknown	360 (13.37)	279 (13.14)	81 (14.24)	
AJCC stage, n (%)				<0.001
I	1178 (43.76)	1081 (50.92)	97 (17.05)	
II	431 (16.01)	360 (16.96)	71 (12.48)	
III	292 (10.85)	191 (9.00)	101 (17.75)	
IV	427 (15.86)	184 (8.67)	243 (42.71)	
Unknown	364 (13.52)	307 (14.46)	57 (10.02)	
T stage, n (%)				<0.001
T1	1268 (47.10)	1153 (54.31)	115 (20.21)	
T2	531 (19.73)	416 (19.59)	115 (20.21)	
T3	279 (10.36)	152 (7.16)	127 (22.32)	
T4	275 (10.22)	123 (5.79)	152 (26.71)	
NR	339 (12.59)	279 (13.14)	60 (10.54)	
N stage, n (%)				<0.001
N0	2143 (79.61)	1826 (86.01)	317 (55.71)	
N1	201 (7.47)	109 (5.13)	92 (16.17)	
N2	202 (7.50)	76 (3.58)	126 (22.14)	
N3	10 (0.37)	2 (0.09)	8 (1.41)	
NR	136 (5.05)	110 (5.18)	26 (4.57)	
M stage, n (%)				<0.001
M0	2549 (94.69)	2050 (96.56)	499 (87.70)	
M1	54 (2.01)	6 (0.28)	48 (8.44)	
NR	89 (3.31)	67 (3.16)	22 (3.87)	
Chemotherapy, n (%)				<0.001
No/unknown	2530 (93.98)	2075 (97.74)	455 (79.96)	
Yes	162 (6.02)	48 (2.26)	114 (20.04)	
Surgery, n (%)				<0.001
No	214 (7.95)	99 (4.66)	115 (20.21)	
Partial excision	1615 (59.99)	1379 (64.96)	236 (41.48)	
Total excision or radical excision	803 (29.83)	601 (28.31)	202 (35.50)	
Unknown type of surgery	60 (2.23)	44 (2.07)	16 (2.81)	
Radiation, n (%)				<0.001
No	1744 (64.78)	1502 (70.75)	242 (42.53)	
Yes	948 (35.22)	621 (29.25)	327 (57.47)	
Combination therapy, n (%)				<0.001
No surgery and no radiotherapy	135 (5.01)	88 (4.15)	47 (8.26)	
Surgery only	1609 (59.77)	1414 (66.60)	195 (34.27)	
Radiotherapy only	79 (2.93)	11 (0.52)	68 (11.95)	
Surgery combined with radiotherapy	869 (32.28)	610 (28.73)	259 (45.52)	
Follow-up, months, M (Q_1_, Q_3_)	93.00 (57.00, 136.00)	107.00 (72.00, 144.00)	27.00 (11.00, 63.00)	<0.001
Survival status, n (%)				<0.001
Overall survival	2123 (78.86)	2123 (100.00)	0 (0.00)	
Cancer specific mortality	341 (12.67)	0 (0.00)	341 (59.93)	
Others mortality	228 (8.47)	0 (0.00)	228 (40.07)	

AJCC, American Joint Committee on Cancer.

### Survival curve of patients with head and neck MEC

**Figure 2:**
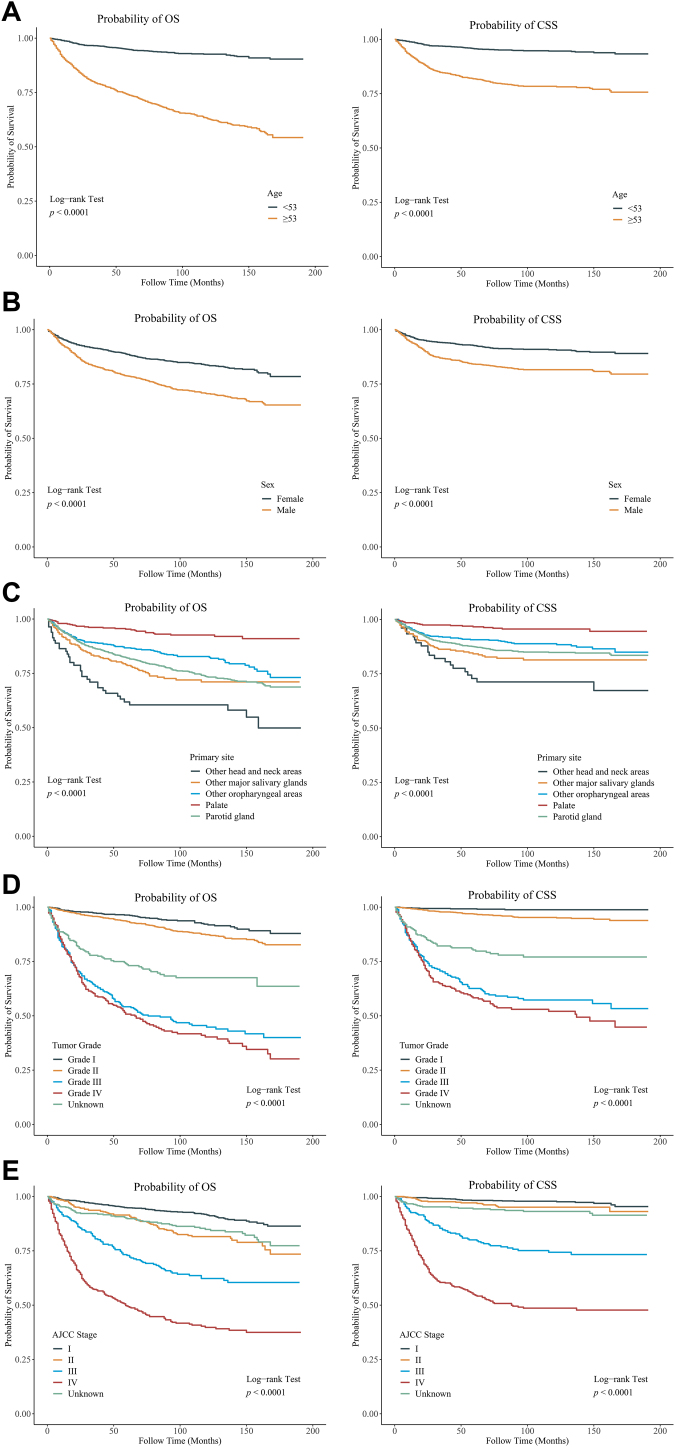
Kaplan-Meier (KM) curves of OS and CSS in patients with head and neck MEC. (A) age subgroups; (B) sex subgroups; (C) tumor primary site subgroups; (D) tumor grade subgroups; (E) AJCC stage subgroups. OS, Overall Survival; CSS, Cancer-Specific Survival; MEC, Mucoepidermoid Carcinoma.

The KM curves of OS and CSS according to age, sex, tumor primary site, tumor grade, and AJCC stage were analyzed. Patients older than 53 years were associated with poorer OS and CSS (*p* < 0.0001) compared to patients younger than 53 years (Fig. 2A). Females had better OS and CSS (*p* < 0.0001) than males (Fig. 2B). Patients with a tumor primary site located in the palate had the best OS and CSS (*p* < 0.0001), while patients with a tumor primary site located in other head and neck areas had the worst OS and CSS (Fig. 2C). Patients with grade I or grade II were related to better OS and CSS (*p* < 0.0001) compared to those with grade III or grade IV (Fig. 2D). Patients with AJCC stage I had optimal OS and CSS (*p* < 0.0001), whereas patients with AJCC stage IV had poorer OS and CSS (Fig. 2E).

### Factors correlated with OS and CSS

Factors correlated with OS and CSS in patients with head and neck MEC were presented in Table 2. For the multivariable analysis of OS, older age (≥53 years), males, unmarried, lower income (<75,000$), tumor site in other head and neck areas, higher tumor grade (III/IV), larger tumor size, and higher AJCC stage were linked to worse OS in patients with head and neck MEC, whereas others race and tumor site in the palate or other oropharyngeal areas were correlated with better OS. In the multivariable analysis of CSS, the factors affecting CSS were consistent with OS.

**Table 2:** Factor associated with OS and CSS in patients with head and neck MEC.

Variables	OS		CSS	
	HR (95%CI)	*p*	HR (95%CI)	*p*
Age				
<53	Ref		Ref	
≥53	5.89 (4.72–7.34)	<0.001	4.42 (3.39–5.76)	<0.001
Sex				
Female	Ref		Ref	
Male	1.91 (1.62–2.26)	<0.001	2.06 (1.65–2.57)	<0.001
Race				
White	Ref		Ref	
Black	0.92 (0.71–1.18)	0.507	0.94 (0.68–1.30)	0.702
Others	0.42 (0.30‒0.60)	<0.001	0.43 (0.28‒0.68)	<0.001
Unknown	0.06 (0.01‒0.46)	0.006	0.11 (0.01‒0.75)	0.025
Marital status				
Married	Ref		Ref	
Unmarried	1.49 (1.25–1.76)	<0.001	1.27 (1.02–1.58)	0.030
Unknown	0.74 (0.51–1.07)	0.109	0.49 (0.28‒0.84)	0.009
Income				
≥75,000$	Ref		Ref	
<75,000$	1.31 (1.09–1.58)	0.004	1.26 (0.99–1.59)	0.056
Primary site				
Parotid gland	Ref		Ref	
Other major salivary glands	1.13 (0.87–1.47)	0.352	1.26 (0.91–1.74)	0.165
Palate	0.27 (0.18‒0.39)	<0.001	0.28 (0.17‒0.47)	<0.001
Other oropharyngeal areas	0.72 (0.58‒0.90)	0.004	0.78 (0.58–1.03)	0.083
Other head and neck areas	1.93 (1.35–2.74)	<0.001	2.08 (1.33–3.27)	0.001
Tumor grade				
I/II	Ref		Ref	
III/IV	7.89 (6.58–9.47)	<0.001	17.66 (13.30–23.45)	<0.001
Unknown	3.79 (2.91–4.94)	<0.001	7.79 (5.39–11.26)	<0.001
Tumor size				
<1 cm	Ref		Ref	
1−2 cm	2.20 (1.29–3.74)	0.004	4.92 (1.52–15.97)	0.008
2−4 cm	6.36 (3.83–10.55)	<0.001	19.91 (6.34–62.48)	<0.001
≥4 cm	16.59 (9.91–27.78)	<0.001	63.62 (20.22–200.22)	<0.001
Unknown	4.95 (2.90–8.46)	<0.001	15.65 (4.87–50.28)	<0.001
AJCC stage				
I/II	Ref		Ref	
III/IV	6.09 (5.06–7.32)	<0.001	16.36 (12.00–22.32)	<0.001
Unknown	1.47 (1.09–1.98)	0.012	2.37 (1.46–3.85)	<0.001

OS, Overall Survival; CSS, Cancer-Specific Survival; MEC, Mucoepidermoid Carcinoma; HR, Hazard Ratio; CI, Confidence Interval; Ref, Reference.

Table 3 shows the impact of treatment modality on survival in patients with head and neck MEC. Patients who received chemotherapy had poorer OS compared with patients who did not (multivariable: [HR = 1.44, 95% CI 1.14–1.84]). For surgery, only patients who did not receive surgery (HR = 3.20, 95% CI 2.45–4.18) had worse OS, while no significant difference was detected between partial and total organ excision on patients’ OS (*p* = 0.729). For radiotherapy, no significant difference in OS was observed between patients with and without radiotherapy (*p* = 0.459). For combination therapy, patients who received radiotherapy only (HR = 3.21, 95% CI 2.27–4.53) or no surgery and no radiotherapy (HR = 2.59, 95% CI 1.83–3.67) were linked to worse OS compared to patients who received surgery only, but no significant difference was detected between surgery only and surgery combined with radiotherapy on patients’ OS (*p* = 0.218). In the multivariable analysis of CSS, patients who received chemotherapy were related to worse CSS compared with patients who did not (HR = 1.41, 95% CI 1.08–1.85). For surgery, patients who did not receive surgery (HR = 4.06, 95% CI 2.88–5.72) were correlated with poorer CSS compared with patients who received partial excision, but no significant difference was detected between partial excision and total excision on patients’ CSS (*p* = 0.070). For radiotherapy, no significant difference was detected in CSS between radiotherapy and non-radiotherapy (*p* = 0.124). For combination therapy, patients who received radiotherapy only (HR = 4.43, 95% CI 2.85–6.86) or no surgery and no radiotherapy (HR = 4.09, 95% CI 2.56–6.55) were related to poorer CSS compared to patients who received surgery only, but no significant difference was detected between surgery only and surgery combined with radiotherapy on patients’ CSS (*p* = 0.069).

**Table 3:** The effect of treatment modality on OS and CSS in patients with head and neck MEC.

Treatment	OS				CSS			
	Univariable		Multivariable		Univariable		Multivariable	
	HR (95%CI)	*p*	HR (95%CI)	*p*	HR (95%CI)	*p*	HR (95%CI)	*p*
Chemotherapy								
No/Unknown	Ref		Ref		Ref		Ref	
Yes	6.50 (5.28–8.00)	<0.001	1.44 (1.14–1.84)	0.003	9.76 (7.71–12.35)	<0.001	1.41 (1.08–1.85)	0.012
Surgery								
No	5.80 (4.64–7.25)	<0.001	3.20 (2.45–4.18)	<0.001	9.72 (7.31–12.93)	<0.001	4.06 (2.88–5.72)	<0.001
Partial excision	Ref		Ref		Ref		Ref	
Total excision or radical excision	1.87 (1.55–2.26)	<0.001	1.04 (0.85–1.27)	0.729	2.96 (2.30–3.82)	<0.001	1.28 (0.98–1.67)	0.070
Unknown type of surgery	1.98 (1.20–3.29)	0.008	1.20 (0.71–2.01)	0.497	2.21 (1.08–4.53)	0.031	1.06 (0.51–2.22)	0.870
Radiation								
No	Ref		Ref		Ref		Ref	
Yes	2.85 (2.41–3.36)	<0.001	0.92 (0.75–1.14)	0.459	5.40 (4.26–6.85)	<0.001	1.25 (0.94–1.65)	0.124
Combination therapy								
No surgery and no radiotherapy	3.81 (2.77–5.24)	<0.001	2.59 (1.83–3.67)	<0.001	7.58 (4.93–11.66)	<0.001	4.09 (2.56–6.55)	<0.001
Surgery only	Ref		Ref		Ref		Ref	
Radiotherapy only	17.72 (13.39–23.44)	<0.001	3.21 (2.27–4.53)	<0.001	41.56 (28.92–59.71)	<0.001	4.43 (2.85–6.86)	<0.001
Surgery combined with radiotherapy	2.75 (2.28–3.31)	<0.001	0.87 (0.70–1.09)	0.218	6.11 (4.59–8.12)	<0.001	1.35 (0.98–1.85)	0.069

OS, Overall Survival; CSS, Cancer-Specific Survival; MEC, Mucoepidermoid Carcinoma; HR, Hazard Ratio; CI, Confidence Interval; Ref, Reference.

Multivariable analysis adjusted in OS and CSS: age, sex, race, marital status, income, primary site, tumor grade, tumor size, AJCC stage, chemotherapy (not adjusted in chemotherapy analysis), surgery (not adjusted in surgery or combination therapy analyses), and radiotherapy (not adjusted in radiation or combination therapy analyses).

### Impact of treatment modality on survival in different populations

**Figure 3:**
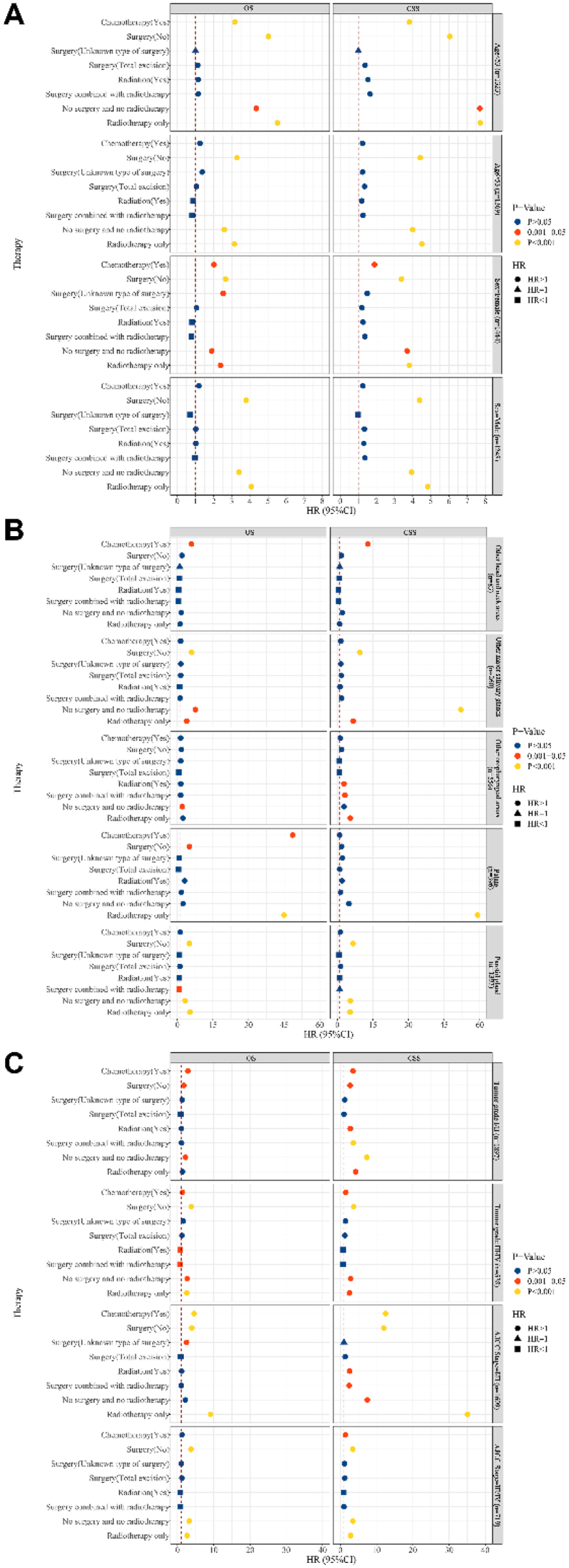
The impact of treatment modality on OS and CSS in different patients. (A) Age and sex subgroups; (B) Tumor primary site subgroups; (C) Tumor grade and AJCC stage subgroups. OS, Overall Survival; CSS, Cancer-Specific Survival; all analyses were multivariable analysis, adjusted for (OS and CSS): age, sex, race, marital status, income, primary site, tumor grade, tumor size, AJCC stage, chemotherapy (not adjusted in chemotherapy analysis), surgery (not adjusted in surgery or combination therapy analyses), and radiotherapy (not adjusted in radiation or combination therapy analyses).

Fig. 3 shows the impact of treatment modality on survival based on age, sex, tumor primary site, tumor grade, and AJCC stage. Patients who received radiotherapy only or no surgery or no surgery and no radiotherapy had poorer OS and CSS at age < 53 years or ≥53 years, and patients who received chemotherapy also had worse OS at age < 53 years (*p* < 0.05) (Fig. 3A). Females or males who received radiotherapy only or no surgery or no surgery and no radiotherapy had poorer OS and CSS, and females who received chemotherapy were also had worse OS (*p* < 0.05) (Fig. 3A). Patients who received radiotherapy only or no surgery or no surgery and no radiotherapy were correlated with worse OS and CSS when the tumor was in the parotid gland and other major salivary glands (*p* < 0.05) (Fig. 3B). Patients who received radiotherapy only were related to poorer OS and CSS (*p* < 0.05). When the tumor was in other head and neck areas, patients who received chemotherapy were linked to worse OS and CSS (*p* < 0.05). Patients who received chemotherapy or no surgery or no surgery and no radiotherapy had worse OS and CSS when the tumor grade was I/II or III/IV, while patients with tumor grade III/IV receiving surgery combined with radiotherapy may be related to better OS (*p* < 0.05) (Fig. 3C). Patients who received radiotherapy only or no surgery had worse OS and CSS when the AJCC stage was I/II or III/IV (*p* < 0.05) (Fig. 3C). In addition, OS and CSS were worse in stage I/II patients who received chemotherapy and in stage III/IV patients who did not receive surgery and radiotherapy (*p* < 0.05).

Moreover, the effects of treatment modality on patients with the same tumor grade and tumor primary site were shown in Supplementary Table 1 and Supplementary Table 2. For patients with parotid gland MEC, patients with tumor grade III/IV who received radiotherapy only or no surgery and no radiotherapy had poorer CSS compared to patients who received surgery only, but patients with tumor grade I/III who received surgery combined with radiotherapy had poorer CSS (*p* < 0.05). For patients with palate MEC, patients with tumor grade I/III who did not receive surgery and radiotherapy had worse CSS compared to patients who received surgery only (*p* < 0.05). For other patients with the same tumor grade and tumor primary site, the stratification of tumor grade and tumor primary site resulted in too small a sample size for the corresponding group to draw conclusions.

## Discussion

We analyzed the factors affecting the survival of head and neck MEC, especially the impact of treatment modalities on patient survival. Older age, males, tumor sites in other head and neck areas, higher tumor grade, and higher AJCC stage were correlated with poorer OS and CSS. For treatment, chemotherapy, no surgery, radiotherapy only, and no surgery and no radiotherapy were linked to worse OS and CSS. In addition, no significant differences were detected between partial excision and total excision on patients’ OS and CSS, as well as between surgery only and surgery combined with radiotherapy.

MEC is categorized into three histological grades of low, intermediate, and high according to the proportions of epidermoid squamous cells, mucinous cells, and intermediate cells.^2^ Head and neck is the most common sites of MEC, and the primary MEC in the parotid gland accounts for the largest proportion. Our results indicated that the percentage of patients with parotid gland MEC was 51.89%, followed by other oropharyngeal areas MEC (20.65%) and palate MEC (14.71%). Survival analysis presented that patient with palate MEC had the best OS and CSS, while patients with other head and neck areas MEC had the worst OS and CSS. Mimica et al. showed that patients with MEC whose tumors were in the hard palate and posterior lobe of the trigeminal nerve had the highest survival rates, while those whose tumors were in the paranasal sinuses and submandibular gland had the lowest survival rates.^16^ Zhou et al. reported a 90% survival rate for patients with palate MEC at 6- to 60-month postoperative follow-up.^17^ Age has a greater impact on survival in MEC patients. A 15-year single-center study showed that age > 56 years (vs. ≤56 years) may be the only independent negative prognostic factor for declining OS.^18^ Our results demonstrated that patients aged ≥ 53 years were correlated with poorer OS and CSS compared to those aged < 53 years. Sex has an impact on both the occurrence and prognosis of head and neck cancer. In our study, the proportion of head and neck MEC was higher in females than in males (53.64% vs. 46.36%), but both OS and CSS were poorer in males than in females. This may be related to the expression of sex hormones between males and females.^19^, ^20^ Aquino et al. demonstrated that the androgen receptor was aberrantly expressed in patients with MEC.^21^ In addition, our results suggested that among patients with different tumor grades or AJCC stages, higher grades or stages were related to worse survival.

Surgical resection with disease-free margins is the mainstay of treatment for MEC.^22^ This study analyzed the impact of different treatment modalities on survival in patients with head and neck MEC. In univariable analysis, patients who did not receive surgery or who received total excision were likened to worse OS and CSS compared to those who received partial excision. However, multivariable analysis demonstrated that patients who did not receive surgery were related to worse OS and CSS compared with partial organ excision, whereas patients who received total organ excision did not present better OS and CSS. The results of univariable analyses may be influenced by a variety of confounders. Moreover, patients who received partial excision may have lower disease severity. Therefore, we performed stratified analyses according to tumor grade and AJCC stage. The same results as the multivariable analyses described above were also shown in patients with different tumor grades and AJCC stages. These results suggested that when total resection is possible, total resection is not necessarily superior to partial resection and may lead to greater trauma and loss of organ function. In the analysis of combined treatment (surgery and radiotherapy), no significant differences were detected between surgery only and surgery combined with radiotherapy on patients’ OS and CSS. However, patients who received radiotherapy only or no surgery and no radiotherapy (vs. surgery only) were correlated with worse OS and CSS. Adjuvant radiotherapy enhances local regional control and is usually applied to patients with high-risk characteristics such as perineural invasion, lymph node involvement, advanced high-grade tumors, positive margins after resection, and extra-glandular extension.^22^, ^23^, ^24^, ^25^ These results indicated that when negative surgical margins can be achieved, surgical treatment remains the primary option for a better patient prognosis. Furthermore, our results found that patients who received chemotherapy had poorer OS and CSS compared to patients who did not. The advantage of systemic therapy in improving survival in patients with head and neck MEC is not clear, and it is often used as an option for inoperable patients with locally advanced disease or as a palliative treatment when the disease is rapidly progressing.^22^, ^26^, ^27^

This study examined the survival characteristics of head and neck MEC patients and their influencing factors based on the SEER database. The effects of treatments on OS and CSS were analyzed and further analyzed according to different ages, sexes, tumor primary sites, tumor grades, and AJCC stages. However, there were several limitations to this study. First, specific doses and courses of radiotherapy and chemotherapy are not available from the SEER database, which may cause some bias in the results. Second, some prognostic factors such as surgical margin status, nutritional status, and perineural invasion could not be accessed. Third, data describing disease recurrence or disease-free survival were not recorded in the SEER database. Fourth, although this study was based on a large sample of the SEER database, there were still some categories with a small number of patients. For example, we combined some rare sites in different tumor primary sites as other head and neck areas. However, there may be heterogeneity among these sites and the results need to be interpreted with caution.

## Conclusions

We investigated the prognostic factors and the effect of different treatments on patients with head and neck MEC. Older age, males, higher tumor grade, and higher AJCC stage were correlated with poorer OS and CSS, while the primary tumor site in the palate was correlated with better OS and CSS. Chemotherapy, no surgery, radiotherapy only, and no surgery and no radiotherapy were related to worse OS and CSS. Furthermore, no significant differences were detected between partial excision and total excision on patients’ OS and CSS, as well as between surgery only and surgery combined with radiotherapy. These results suggested that surgical resection only may be a better survival option for patients with head and neck MEC.

## Declarations

Ethics approval and consent to participate: Not applicable.

Consent for publication: Not applicable.

Availability of data and materials: The datasets used and/or analyzed during the current study available from the corresponding author on reasonable request.

## Authors’ contributions

(1) Lin Gui: Conceiving and designing the study. (2) Lin Gui, Yiming Zhu, Ye Zhang, Le Tang, Jiarui Yao: Collecting the data. (3) Lin Gui, Yiming Zhu, Ye Zhang, Le Tang, Jiarui Yao: Analyzing and interpreting the data. (4) Lin Gui: Writing the manuscript. (5) Lin Gui: Providing critical revisions that are important for the intellectual content. (6) Lin Gui, Yiming Zhu, Ye Zhang, Le Tang, Jiarui Yao: Approving the final version of the manuscript.

## Funding

This study was supported by the Beijing Hope Run Special Fund of Cancer Foundation of China (nº LC2022A30) and the Major Project of Medical Oncology Key Foundation of Cancer Hospital Chinese Academy of Medical Sciences (nº CICAMS-MOMP2022010).

## Conflicts of interest

The authors declare no have conflicts of interest.

This research did not receive any specific grant from funding agencies in the public, commercial, or not-for-profit sectors.
