# Supplementary material for: Epidemiology, prognostic factors, and treatment of head and neck mucoepidermoid carcinoma: Analysis of the surveillance, epidemiology, and end results database
Source: Braz J Otorhinolaryngol. 2024 Jun 8;90(5):101450. doi: 10.1016/j.bjorl.2024.101450 (PMC11324812; doi:10.1016/j.bjorl.2024.101450)
Supplement: Supplementary file 1 [file mmc1.docx]

BJORL-D-24-00043_Supplementary Material

**Supplement Table 1** Effect of treatment modality on OS and CSS in patients with tumor grade I/II MEC of the head and neck.

| **Tumor primary sites** | **Variables** | **OS** | | **CSS** | |
| --- | --- | --- | --- | --- | --- |
|  |  | **HR (95%CI)** | ***p*** | **HR (95%CI)** | ***p*** |
| Other head and neck areas (n = 30) | Chemotherapy |  |  |  |  |
|  | No/Unknown | Ref |  | Ref |  |
|  | Yes | 0.03 (0.00‒14312611) | 0.728 | ‒ | 1.000 |
|  | Surgery |  |  |  |  |
|  | No | ‒ | 0.999 | ‒ | 1.000 |
|  | Partial excision | Ref |  | Ref |  |
|  | Total excision or radical excision | 726.28 (0.14‒3712168) | 0.131 | ‒ | 1.000 |
|  | Radiation |  |  |  |  |
|  | No | Ref |  | Ref |  |
|  | Yes | ‒ | 0.425 | ‒ | 1.000 |
|  | Combination therapy |  |  |  |  |
|  | Surgery combined with radiotherapy | 0.01 (0.00‒51.86) | 0.284 | ‒ | 1.000 |
|  | No surgery and no radiotherapy | ‒ | 1.000 | ‒ | 1.000 |
|  | Radiotherapy only | 3.69 (0.00‒.) | 1.000 | ‒ | 1.000 |
|  | Surgery only | Ref |  | Ref |  |
| Other major salivary glands (n = 162) | Chemotherapy |  |  |  |  |
|  | No/Unknown | Ref |  | Ref |  |
|  | Yes | 13.72 (0.40‒473.90) | 0.147 | ‒ | 0.999 |
|  | Surgery |  |  |  |  |
|  | None | 2.73 (0.12‒62.84) | 0.530 | ‒ | 0.999 |
|  | Partial excision | Ref |  | Ref |  |
|  | Total excision or radical excision | 3.35 (0.93‒12.10) | 0.065 | ‒ | 0.999 |
|  | Radiation |  |  |  |  |
|  | No | Ref |  | Ref |  |
|  | Yes | 1.14 (0.29‒4.51) | 0.848 | ‒ | 0.999 |
|  | Combination therapy |  |  |  |  |
|  | Surgery combined with radiotherapy | 1.32 (0.32‒5.36) | 0.699 | 4.45 (0.16‒123.50) | 0.379 |
|  | No surgery and no radiotherapy | ‒ | 0.999 | ‒ | 1.000 |
|  | Radiotherapy only | 5.00 (0.09‒271.32) | 0.429 | 0.70 (0.00‒128.07) | 0.891 |
|  | Surgery only | Ref |  | Ref |  |
| Other oropharyngeal areas (n = 432) | Chemotherapy |  |  |  |  |
|  | No/Unknown | Ref |  | Ref |  |
|  | Yes | 5.80 (1.59‒21.14) | 0.008 | 2.03 (0.30‒13.84) | 0.470 |
|  | Surgery |  |  |  |  |
|  | No | 1.27 (0.51‒3.15) | 0.604 | 1.11 (0.23‒5.45) | 0.898 |
|  | Partial excision | Ref |  | Ref |  |
|  | Total excision or radical excision | 0.40 (0.13‒1.25) | 0.115 | 0.28 (0.06‒1.31) | 0.105 |
|  | Radiation |  |  |  |  |
|  | No | Ref |  | Ref |  |
|  | Yes | 1.15 (0.48‒2.73) | 0.758 | 8.32 (1.84‒37.62) | 0.006 |
|  | Combination therapy |  |  |  |  |
|  | Surgery combined with radiotherapy | 0.93 (0.39‒2.18) | 0.860 | 4.98 (1.18‒21.14) | 0.029 |
|  | No surgery and no radiotherapy | 1.42 (0.47‒4.22) | 0.534 | ‒ | 0.997 |
|  | Radiotherapy only | 1.57 (0.32‒7.74) | 0.581 | 13.70 (1.75‒107.46) | 0.013 |
|  | Surgery only | Ref |  | Ref |  |
| Palate (n = 340) | Chemotherapy |  |  |  |  |
|  | No/Unknown | Ref |  | Ref |  |
|  | Surgery |  |  |  |  |
|  | None | 5.65 (1.16‒27.54) | 0.032 | 8.63 (0.93‒80.11) | 0.058 |
|  | Partial excision | Ref |  | Ref |  |
|  | Total excision or radical excision | 1.05 (0.26‒4.19) | 0.948 | 1.68 (0.21‒13.62) | 0.629 |
|  | Radiation |  |  |  |  |
|  | No | Ref |  | Ref |  |
|  | Yes | 2.52 (0.58‒11.02) | 0.220 | 1.54 (0.21‒11.27) | 0.672 |
|  | Combination therapy |  |  |  |  |
|  | Surgery combined with radiotherapy | 2.88 (0.51‒16.22) | 0.230 | 2.54 (0.29‒22.01) | 0.398 |
|  | No surgery and no radiotherapy | 6.59 (0.96‒45.08) | 0.054 | 11.80 (1.06‒131.22) | 0.045 |
|  | Radiotherapy only | 11.16 (0.82‒152.44) | 0.070 | ‒ | 1.000 |
|  | Surgery only | Ref |  | Ref |  |
| Parotid gland (n = 933) | Chemotherapy |  |  |  |  |
|  | No/Unknown | Ref |  | Ref |  |
|  | Yes | 2.34 (0.97‒5.67) | 0.059 | 4.10 (1.30‒12.97) | 0.016 |
|  | Surgery |  |  |  |  |
|  | None | 2.39 (0.90‒6.36) | 0.081 | 7.69 (1.80‒32.83) | 0.006 |
|  | Partial excision | Ref |  | Ref |  |
|  | Total excision or radical excision | 0.86 (0.53‒1.38) | 0.530 | 1.48 (0.60‒3.68) | 0.398 |
|  | Radiation |  |  |  |  |
|  | No | Ref |  | Ref |  |
|  | Yes | 0.97 (0.61‒1.52) | 0.878 | 2.63 (1.10‒6.30) | 0.030 |
|  | Combination therapy |  |  |  |  |
|  | Surgery combined with radiotherapy | 1.05 (0.67‒1.67) | 0.821 | 4.80 (1.73‒13.31) | 0.003 |
|  | No surgery and no radiotherapy | 4.14 (1.55‒11.07) | 0.005 | 45.89 (9.77‒215.51) | <0.001 |
|  | Radiotherapy only | ‒ | 0.995 | ‒ | 0.998 |
|  | Surgery only | Ref |  | Ref |  |

OS, Overall Survival; CSS, Cancer-Specific Survival; MEC, Mucoepidermoid Carcinoma; HR, Hazard Ratio; CI, Confidence Interval; Ref, Reference.

Multivariable analysis adjusted in OS and CSS: age, sex, race, marital status, income, tumor size, AJCC stage, chemotherapy (not adjusted in chemotherapy analysis), surgery (not adjusted in surgery or combination therapy analyses), and radiotherapy (not adjusted in radiation or combination therapy analyses).

**Supplement Table 2** Effect of treatment modality on OS and CSS in patients with tumor grade III/IV MEC of the head and neck.

| **Tumor primary sites** | **Variables** | **OS** |  | **CSS** | ***p*** |
| --- | --- | --- | --- | --- | --- |
|  |  | **HR (95%CI)** | ***p*** | **HR (95%CI)** |  |
| Other head and neck areas (n = 35) | Chemotherapy |  |  |  |  |
|  | No/Unknown | Ref |  | Ref |  |
|  | Yes | 7.07 (1.47‒33.99) | 0.015 | 12.24 (1.87‒80.00) | 0.009 |
|  | Surgery |  |  |  |  |
|  | No | 1.43 (0.20‒10.00) | 0.718 | 0.57 (0.04‒8.82) | 0.691 |
|  | Partial excision | Ref |  | Ref |  |
|  | Total excision or radical excision | 0.98 (0.22‒4.37) | 0.976 | 0.84 (0.13‒5.22) | 0.850 |
|  | Radiation |  |  |  |  |
|  | No | Ref |  | Ref |  |
|  | Yes | 0.35 (0.07‒1.79) | 0.209 | 0.39 (0.06‒2.50) | 0.320 |
|  | Combination therapy |  |  |  |  |
|  | Surgery combined with radiotherapy | 0.41 (0.07‒2.56) | 0.341 | 0.27 (0.03‒2.41) | 0.239 |
|  | No surgery and no radiotherapy | 2.26 (0.12‒42.21) | 0.585 | 0.21 (0.00‒9.92) | 0.428 |
|  | Radiotherapy only | 0.50 (0.08‒3.00) | 0.445 | 0.27 (0.03‒2.68) | 0.263 |
|  | Surgery only | Ref |  | Ref |  |
| Other major salivary glands (n = 62) | Chemotherapy |  |  |  |  |
|  | No/Unknown | Ref |  | Ref |  |
|  | Yes | 1.45 (0.51‒4.10) | 0.481 | 2.14 (0.73‒6.25) | 0.163 |
|  | Surgery |  |  |  |  |
|  | No | 23.48 (3.72‒148.05) | <0.001 | 24.43 (3.63‒164.24) | 0.001 |
|  | Partial excision | Ref |  | Ref |  |
|  | Total excision or radical excision | 1.13 (0.44‒2.90) | 0.794 | 1.34 (0.54‒3.35) | 0.529 |
|  | Radiation |  |  |  |  |
|  | No | Ref |  | Ref |  |
|  | Yes | 0.71 (0.21‒2.34) | 0.569 | 0.78 (0.26‒2.31) | 0.650 |
|  | Combination therapy |  |  |  |  |
|  | Surgery combined with radiotherapy | 0.95 (0.31‒2.92) | 0.923 | 0.93 (0.30‒2.89) | 0.906 |
|  | No surgery and no radiotherapy | ‒ | ‒ | ‒ | . |
|  | Radiotherapy only | 37.28 (5.04‒275.53) | <.001 | 9.19 (0.81‒104.13) | 0.073 |
|  | Surgery only | Ref |  | Ref |  |
| Other oropharyngeal areas (n = 73) | Chemotherapy |  |  |  |  |
|  | No/Unknown | Ref |  | Ref |  |
|  | Yes | 2.06 (0.79‒5.37) | 0.139 | 2.64 (0.97‒7.21) | 0.059 |
|  | Surgery |  |  |  |  |
|  | No | 1.54 (0.58‒4.11) | 0.387 | 1.39 (0.46‒4.19) | 0.561 |
|  | Partial excision | Ref |  | Ref |  |
|  | Total excision or radical excision | 1.14 (0.43‒3.02) | 0.798 | 1.60 (0.54‒4.75) | 0.395 |
|  | Radiation |  |  |  |  |
|  | No | Ref |  | Ref |  |
|  | Yes | 0.66 (0.24‒1.78) | 0.410 | 0.66 (0.21‒2.10) | 0.483 |
|  | Combination therapy |  |  |  |  |
|  | Surgery combined with radiotherapy | 0.84 (0.27‒2.65) | 0.764 | 0.83 (0.22‒3.11) | 0.783 |
|  | No surgery and no radiotherapy | 1.71 (0.38‒7.70) | 0.483 | 1.17 (0.21‒6.61) | 0.857 |
|  | Radiotherapy only | 1.00 (0.23‒4.36) | 1.000 | 0.81 (0.15‒4.25) | 0.803 |
|  | Surgery only | Ref |  | Ref |  |
| Palate (n = 23) | Chemotherapy |  |  |  |  |
|  | No/Unknown | Ref |  | Ref |  |
|  | Yes | ‒ | 1.000 | ‒ | 1.000 |
|  | Surgery |  |  |  |  |
|  | No | ‒ | 1.000 | ‒ | 1.000 |
|  | Partial excision | Ref |  | Ref |  |
|  | Total excision or radical excision | ‒ | 1.000 | ‒ | 1.000 |
|  | Radiation |  |  |  |  |
|  | No | Ref |  | Ref |  |
|  | Yes | ‒ | 0.999 | ‒ | 0.999 |
|  | Combination therapy |  |  |  |  |
|  | Surgery combined with radiotherapy | ‒ | 0.999 | ‒ | 1.000 |
|  | Radiotherapy only | ‒ | 0.999 | ‒ | 0.999 |
|  | Surgery only | Ref |  | Ref |  |
| Parotid gland (n = 345) | Chemotherapy |  |  |  |  |
|  | No/Unknown | Ref |  | Ref |  |
|  | Yes | 1.09 (0.75‒1.58) | 0.642 | 1.21 (0.81‒1.81) | 0.352 |
|  | Surgery |  |  |  |  |
|  | No | 8.79 (4.87‒15.87) | <0.001 | 8.18 (4.25‒15.73) | <0.001 |
|  | Partial excision | Ref |  | Ref |  |
|  | Total excision or radical excision | 1.30 (0.94‒1.80) | 0.118 | 1.31 (0.88‒1.94) | 0.183 |
|  | Radiation |  |  |  |  |
|  | No | Ref |  | Ref |  |
|  | Yes | 0.68 (0.48‒0.96) | 0.027 | 0.73 (0.48‒1.10) | 0.135 |
|  | Combination therapy |  |  |  |  |
|  | Surgery combined with radiotherapy | 0.57 (0.40‒0.81) | 0.002 | 0.64 (0.41‒1.00) | 0.051 |
|  | No surgery and no radiotherapy | 3.07 (1.27‒7.46) | 0.013 | 3.87 (1.47‒10.18) | 0.006 |
|  | Radiotherapy only | 6.25 (3.32‒11.77) | <0.001 | 5.53 (2.67‒11.43) | <0.001 |
|  | Surgery only | Ref |  | Ref |  |

OS, Overall Survival; CSS, Cancer-Specific Survival; MEC, Mucoepidermoid Carcinoma; HR, Hazard Ratio; CI, Confidence Interval; Ref, Reference.

Multivariable analysis adjusted in OS and CSS: age, sex, race, marital status, income, tumor size, AJCC stage, chemotherapy (not adjusted in chemotherapy analysis), surgery (not adjusted in surgery or combination therapy analyses), and radiotherapy (not adjusted in radiation or combination therapy analyses).
